# *Mentha piperita* Supplementation Promotes Growth, Immunity, and Disease Resistance in Nile tilapia Against *Aeromonas hydrophila*

**DOI:** 10.3390/pathogens14040378

**Published:** 2025-04-12

**Authors:** Attia A. Abou Zaid, Nagwa H. Mohammed, Ahmed E. Elshafey, Ebtehal E. Hussein, Adel M. El-Gamal, Haitham G. Abo-Al-Ela

**Affiliations:** 1 Department of Aquaculture, Faculty of Aquatic and Fisheries Sciences, Kafrelsheikh University, Kafrelsheikh 33516, Egyptahmed_alshafei@fsh.kfs.edu.eg (A.E.E.); 2 Key Laboratory of Freshwater Aquatic Genetic Resources, Ministry of Agriculture and Rural Affairs, Shanghai Ocean University, Shanghai 201306, China; 3 Key Laboratory of Exploration and Utilization of Aquatic Genetic Resources, Ministry of Education, Shanghai Ocean University, Shanghai 201306, China; 4 Poultry and Fish Production Department, Faculty of Agriculture, Menoufia University, Shebin El-Kom 32516, Egypt; 5 Unit of Bacteriology, Animal Health Research Institute, Kafrelsheikh Branch, Agricultural Research Center, Kafrelsheikh 33511, Egypt; 6 Genetics and Biotechnology, Department of Aquaculture, Faculty of Fish Resources, Suez University, Suez 43221, Egypt

**Keywords:** *Aeromonas hydrophila*, disease resistance, immunity, *Mentha piperita*, *Oreochromis niloticus*

## Abstract

This study investigated the effects of dietary supplementation with *Mentha piperita* (MP) on growth, immune enhancement, and disease resistance in Nile tilapia (*Oreochromis niloticus*) over a 90-day period, particularly against *Aeromonas hydrophila*. MP was incorporated into the diets at concentrations of 0.0%, 0.2%, 0.4%, and 0.6%. Analysis of the essential oil composition of MP identified menthol derivatives as the primary components, along with other bioactive compounds. The results revealed that MP supplementation significantly enhanced growth performance, with fish receiving the 0.6% MP diet achieving the highest weight gain, growth rate, and feed efficiency. Additionally, MP significantly enhanced the fish’s resistance to *A. hydrophila* infection, with the highest survival rate observed in the 0.6% MP group. Further analyses revealed that MP positively influenced blood parameters, improving RBC and WBC counts, hemoglobin levels, as well as serum immunoglobulin M and phagocytic activity. MP also mitigated oxidative stress by increasing antioxidant enzyme activity and reducing malondialdehyde levels. Moreover, MP supplementation at the concentration of 0.6% maintained intestinal integrity against bacterial damage. Gene expression analysis showed that MP upregulated insulin-like growth factor 1, suggesting a potential mechanism for improved growth. Interestingly, MP downregulated the expression of the inflammatory gene nuclear factor kappa B before the bacterial challenge, while its expression remained more downregulated post-challenge compared to control. These findings highlight the potential of MP as an effective feed additive that enhances growth rates in Nile tilapia, boosts immunity against diseases, and improves their overall health.

## 1. Introduction

The farming of aquatic organisms, including water-accommodating animals and plants, is generally termed aquaculture, which occurs in both marine and freshwater systems [1]. While marine aquaculture often receives more attention for its role in seafood and aquaculture production, freshwater aquaculture is crucial for global food security and economic growth [2]. Freshwater aquaculture involves farming various aquatic species such as fish, crustaceans, mollusks, and aquatic plants in inland water bodies, including fishponds, natural or man-made lakes, rivers, and recirculation systems [3]. This sector is an important source of income, protein, and other nutrients, especially in areas with restricted access to marine products [4]. Species, such as tilapias, catfish, carps, and trout, commonly cultured in freshwater systems, are easier to obtain and cheaper than marine species, playing a crucial role in the distribution chain for local communities and small-scale farmers [1].

The tilapia, a group of cichlid fish mainly found in Africa, is among the most common species in aquaculture [5]. It reproduces quickly, can be raised in various conditions, and its production is relatively inexpensive, making it a popular choice in fish farming worldwide [6]. Tilapia is an essential source of protein and other nutrients for humans, particularly in the middle- and low-income countries. It has a relatively good taste, and its affordability makes it accessible to many consumers [7]. However, diseases remain a significant concern in tilapia farming. Bacterial diseases such as *Aeromonas* and *Streptococcus* species cause high mortality rates and economic losses [8]. In addition, parasitic infestations—such as those caused by *Diplostomum* and *Contracaecum* species—and viral infections, including Tilapia lake virus and Tilapia larvae encephalitis virus, also negatively affect tilapia health and overall productivity [9,10].

*Aeromonas hydrophila* is an opportunistic pathogen commonly found in freshwater environments. It can affect a wide range of freshwater fish, as well as other aquatic animals such as soft-shelled turtles. When infections occur, they can result in high mortality rates in both wild and farmed species, including catfish and tilapia [11,12]. *A. hydrophila* employs various mechanisms to evade the host immune system. For example, it produces specific nucleases (e.g., *ahn*) that have been shown to influence its susceptibility to being killed by fish macrophages [13]. However, an overactive host immune response can exacerbate the damage caused by *A. hydrophila*. For instance, excessive expression of tumor necrosis factor α (*tnfα*) amplifies the pathogen’s harmful effects on the liver—such as necrosis, cell swelling, nuclear displacement, and blurred cellular boundaries. It also intensifies the inflammatory response in the midgut, leading to increased villi rupture, vacuolization, fusion, and a marked reduction in goblet cell numbers. These changes collectively result in severe intestinal barrier damage [14].

Additionally, antibiotic resistance is frequently observed in bacteria isolated from fish, including *A. hydrophila* [11,15]. This has prompted research into alternative medicinal strategies to enhance immune responses, improve the antibacterial activity of antibiotics—potentially through synergistic mechanisms—and strengthen overall disease resistance [16,17]. Studies have shown that medicinal herbs play a positive modulatory role in fish, supporting growth, immune function, and reproductive health [18]. These herbs influence key effectors of growth (e.g., growth hormone (Gh) and insulin-like growth factor 1 (Igf1)), immunity (e.g., mucin-like protein, interleukin (Il) 1β, Tnfα, Il6, and interferon γ), and reproduction (e.g., vitellogenins, androgen receptors, estrogen receptors, and follicle-stimulating hormone β) [18].

Studies indicate that natural immunostimulants, including certain medicinal plants, probiotics, and other compounds, can enhance both the innate and adaptive immunity of fish against infections [19,20,21]. Plants that are rich in bioactive compounds—such as essential oils, polysaccharides, and polyphenols—serve as effective natural immunostimulants [22]. Dietary peppermint (*Mentha piperita*; MP) can improve tilapia resistance to bacterial and parasitic infections [23,24]. Plant-derived immunostimulants are eco-friendly and help reduce antibiotic use, controlling the emergence of antibiotic-resistant bacteria.

MP, known for its stimulating aroma and taste, has been shown to function as a natural immunostimulant in tilapia aquaculture, enhancing disease resistance against various pathogens, including bacteria (e.g., *Vibrio* species) [23,25]. MP has also demonstrated effective antiviral activity and notable free-radical scavenging properties, attributed to its high content of phenolic acids and flavonoids [26]. It can modulate the levels of key pro-inflammatory mediators and cytokines, including nitric oxide, TNFα, IL6, and prostaglandin E2 [26].

MP contains bioactive compounds such as menthol, menthofuran, menthyl acetate, menthone, and phenolic compounds with antioxidant and anti-inflammatory properties [27,28]. These components improve the fish’s immune system in multiple ways: they increase phagocytosis, activating cells that patrol and eliminate pathogens [29]; they stimulate antibody synthesis, helping identify and neutralize pathogens [30]; and they regulate cytokine production, orchestrating the immune response [29]. Additionally, peppermint’s antioxidative properties protect immune cells from oxidation, maintaining their functionality [31]. Tilapia fed with peppermint-supplemented diets gained better protection against bacterial infections, such as *Vibrio alginolyticus*, improving their immunity and other qualities that enhance their ability to fight infections [23]. Moreover, peppermint can elevate the fish’s blood parameters, including red and white blood cell counts, further fortifying their overall health and ability to combat infections [32]. Peppermint’s eco-friendliness compared to synthetic antibiotics, which are expensive and environmentally harmful, makes it a preferred immunostimulant [33].

Several studies have investigated the effects of MP on Nile tilapia (*Oreochromis niloticus*), focusing on its impact against parasites [24], *Vibrio alginolyticus* [23], *Streptococcus agalactiae* [34], as well as its role as a stress mitigator during fish transportation [35]. Additionally, MP has been studied in combination with probiotics to enhance resistance against *Aeromonas hydrophila* in *Catla catla* (Hamilton, 1822) [36]. These studies examined various parameters to understand the mechanisms by which MP influences fish physiological systems.

Previous studies have often tested MP leaves in high doses, ranging from 1% to 8% of the diet in various fish species [23,25,36,37]. In Nile tilapia, experiments using MP leaf concentrations of 0%, 2%, 3%, and 4% found that 2% was the most suitable level [23]. However, these concentrations may be considered feed raw materials rather than feed additives. Additionally, high doses of MP could pose potential risks [38]. For example, pulegone, a component of peppermint oil, should not exceed a 1% concentration in external preparations due to its potential toxic effects. High doses can also lead to hepatotoxicity [39]. In fish, the optimal supplementation levels remain unclear, and excessive concentrations may cause adverse health effects, including stress and digestive issues [27]. Therefore, lower concentrations of MP should be tested as a feed additive to evaluate both its production and health benefits. Moreover, lower doses may offer greater potential for long-term inclusion compared to higher doses.

Therefore, further research is needed to clarify the mechanisms of MP at relatively lower concentrations in different fish species and to explore its potential benefits against other, yet unstudied, pathogens. This study aimed to evaluate the effects of incorporating MP into Nile tilapia diets at low doses (0.0%, 0.2%, 0.4%, and 0.6%). Key areas of investigation included growth performance, survival rates, blood parameters, immune responses, oxidative stress markers, and intestinal health, particularly following an *A. hydrophila* challenge, a significant pathogen in aquaculture. Additionally, the study analyzed the composition of *M. piperita* essential oils.

## 2. Materials and Methods

### 2.1. Gas Chromatography and Mass Spectrometry (GC-MS) of Mentha Piperita

The *Mentha piperita* (MP) leaves were subjected to extraction using solid-phase microextraction (SPME) at 50 °C for 20 min before being injected into the GC. The GC-MS system (Agilent Technologies, Inc., Santa Clara, CA, USA) consisted of a 7890B gas chromatograph coupled with a 5977A mass spectrometer detector. The gas chromatograph utilized an HP-5MS column (30 m × 0.25 mm internal diameter, 0.25 μm film thickness). Hydrogen was used as the carrier gas at a flow rate of 1.1 mL/min in splitless injection mode. The temperature program was as follows: an initial temperature of 50 °C (held for 0 min), increasing at a rate of 5 °C/min to 200 °C (held for 0 min), followed by a rise of 20 °C/min to 280 °C, which was maintained for 6 min. The injector and detector temperatures were set at 250 °C and 320 °C, respectively. Mass spectra were obtained using electron ionization (EI) at 70 eV, with a scan range of *m/z* 50–600 and no solvent delay. The ion source temperature was maintained at 230 °C, while the quadrupole temperature was set at 150 °C. Identification of constituents was performed by comparing spectral fragmentation patterns with those in the Wiley and NIST Mass Spectral Library databases.

### 2.2. Experimental Diets and Fish Grouping

Peppermint was purchased from a local market in the Kafrelsheikh district, Egypt, and was identified as MP in the botanical laboratory of the Faculty of Aquatic and Fisheries Sciences, Kafrelsheikh University, Egypt. The leaves of MP were shade-dried, crushed into powder using an electric grinder, and mixed directly with fish feed to achieve three concentrations at 0.2%, 0.4%, and 0.6% of feed. The control diet was prepared without MP (Table 1). The produced pellets were air-dried and kept at 4 °C until use. The chemical composition of the formulated diets was determined according to AOAC [40].

Initially, 180 all-male Nile tilapia (*Oreochromis niloticus*) were procured from a privately owned aquafarm in the Kafrelsheikh district, Egypt. An additional group of fish was reared under the same experimental conditions to determine the challenge adjustment dose and serve as a negative control during the challenge procedures. All fish underwent a week-long acclimatization period in holding containers equipped with adequate oxygenation and submerged filters.

Water parameters were continuously monitored to ensure optimal rearing conditions. Thereafter, uniform-sized fish averaging 10.85 ± 0.096 g were randomly assigned to twelve glass aquariums (four clusters separated into triplicate sets), each housing them in 60 L volumes (15 organisms per container). Every aquarium received consistent airflow, with partial water replacements executed daily using dechlorinated water. Prepared feeds were distributed equally among the tanks at a dose equivalent to 5% of fish weights, administered three times daily (09:00 am, 12:00 pm, and 03:00 pm) for ninety consecutive days (12 h light cycle balanced against 12 h darkness). Body weights were documented routinely every fourteen days to readjust food consumption rates while monitoring the general well-being of the population. Throughout the experiment, water quality and rearing conditions were closely monitored. The water indices had optimal levels of dissolved oxygen at 6.08 ± 0.18 mg/L, temperature at approximately 27 ± 1.18 °C, pH at 7.2 ± 0.16, and ammonia levels ranging from 0.03 to 0.42 mg/L.

### 2.3. Growth Performance and Feed Utilization

At the end of the experiment, fish were anesthetized with tricaine methane sulfonate (MS222, 25 mg/L, Argent Laboratories, Redmond, Washington) to measure the individual weight and length (L) of each fish. Fish growth performance and somatic indices were estimated [42,43]. The other growth performance parameters and feed utilization were calculated as follows:

Weight gain ratio (WG%) = (*W*1 − *W*0)/*W*0 × 100

Feed conversion ratio (FCR) = feed intake (g)/BWG (g)

Specific growth rate (SGR%/day) = 100 × (*lnW*1 − *lnW*0)/*t*

Survival rate (%) = (total number of fish at the end of the experiment/total number of fish at the start of the experiment) × 100

Average daily gain (ADG; g fish^−1^ day^−1^) = *Wt* − *W*0/days

Length gain (cm fish^−1^) = *Lt* − *L*0

Condition factor (K) = 100 × (*W*1/*Lt^3^*)

where *ln* = natural log, *W*1 = final weight at the end of the experiment (g), *W*0 = initial weight (g), *Lt* = final length of fish (cm), *L*0 = initial fish length, and *t* = experimental period (days).

### 2.4. Experimental Bacterial Challenge

Following the 90-day feeding trial, a health assessment was conducted to check for active systemic infections, including those caused by *Aeromonas* spp. For the microbiological examination, blood and tissue samples were collected from three fish per container (liver, spleen, and kidneys). The sampling was performed using the procedures described in Section 2.5: Tissue Sampling and Blood Collection. The collected samples were promptly cultured on general nutrient and selective media to detect bacterial growth. If bacterial growth was detected, biochemical testing, including the VITEK system, was used for identification. Based on the microbiological analysis, the absence of external clinical signs of disease, there being no observed mortalities during the feeding trial, and the healthy appearance of the fish, it was concluded that the fish were in good health and showed no signs of active systemic infection.

After this assessment, the fish were subjected to a bacterial challenge with *Aeromonas hydrophila* (ATCC-13037), obtained from the Microbiological Resources Centre (Cairo Mircen). Before the challenge, an LD50 trial was conducted using fish from the same batch as those used in the 90-day feeding trial. These fish were maintained under identical rearing conditions. For the LD50 assay, fish were injected intraperitoneally with bacterial suspensions at concentrations of 10^4^, 10^5^, 10^6^, 10^7^, 10^8^, 10^9^, and 10^10^ CFU/mL. Mortality was recorded over a 96-h period, and the LD50 value was calculated using probit analysis, yielding an LD50 of 1 × 10^9^ CFU/mL. Based on these results, a bacterial concentration of 1 × 10^8^ CFU/mL (equivalent to 1/10 of the LD50, or sublethal dose) was selected for the subsequent challenge experiment. This sublethal concentration was selected to monitor physiological changes across groups while avoiding high mortality rates that could obscure infection mechanisms, enabling the study of host responses without overwhelming the body’s systems.

The bacterial cells were harvested by centrifugation at 3000× *g* for 10 min, washed twice with sterile phosphate-buffered saline (PBS) to remove residual culture media, and resuspended in PBS before injection. The different experimental groups (in three replicates) then received an intraperitoneal injection of 0.2 mL of a suspension containing 1 × 10⁸ CFU/mL of *A. hydrophila*, as described by Moustafa et al. [44]. Mortality was monitored over a 14-day observation period according to the methods outlined by Naiel et al. [45]. The fish continued to receive their designated diets during this period. Cumulative survival rate was calculated using the following formula:

$$Survival\left(\%\right)=(Number\;of\;fish\;surviving\;at\;the\;end\;of\;the\;\frac{challenge}{Total}\;number\;of\;fish\;at\;the\;challenge\;onset)\times 100$$

At the onset of the challenge, each group consisted of 10 fish per replicate, totaling 30 fish per group.

### 2.5. Tissue Sampling and Blood Collection

Pre- and post-challenge samples were obtained from three fish per tank (nine per group) after 90 days of experimental feeding and 15 days after bacterial challenge. Whole blood was collected from the caudal vein using sterile syringes containing heparin. For serum collection, blood was drawn into plain tubes (without anticoagulants). The collected sera were then stored at −20 °C until further analysis. Liver samples were also taken and kept at −80 °C for RNA extraction. Additionally, intestine samples—including the anterior (immediately following the stomach), middle (central intestinal region), and posterior (preceding the anus) sections—were collected for histopathological examination, as described by Olsson [46] and Okuthe and Bhomela [47].

### 2.6. Hematology and Blood Biochemical Analyses

An automated blood cell counter provided measurements for red blood cells (RBCs), hemoglobin, and packed cell volume (PCV). White blood cell (WBC) counts were determined using a combination of blood smear analysis and hemocytometer data [48]. Differential WBC counts involved preparing and staining blood smears with a modified Wright’s stain. Under high magnification (×100 oil immersion), 100 cells were counted to differentiate percentages of heterophils, lymphocytes, and monocytes. Further analyses included total protein using a commercial kit, albumin using the bromocresol green binding method, calculated globulin, creatinine (colorimetric method as per Heinegård and Tiderström [49]), activities of alanine aminotransferase (ALT) and aspartate aminotransferase (AST) (colorimetric method at 540 nm as described by Diab, et al. [50]), and serum triglycerides and total cholesterol measured using the kits by BioDiagnostic Co., Cairo, Egypt.

### 2.7. Immune and Oxidative Stress Responses

Phagocytic activity and the phagocytic index, as described by Abo-Al-Ela et al. [51], were determined. Briefly, fresh blood samples were incubated with *Candida albicans* at 37 °C for 1 h. Blood smears were prepared, stained with Giemsa, and examined under a microscope. Phagocytic activity represents the percentage of phagocytic cells containing yeast, while the phagocytic index indicates the average number of yeast particles per phagocytic cell.

ELISA, a technique described by Demers and Bayne [52], was used to measure serum lysozyme activity and immunoglobulin M (IgM) levels. A commercially available ELISA kit (BioDiagnostic Co., Egypt) was employed to assess the activities of superoxide dismutase (SOD), glutathione peroxidase (GPx), and catalase (CAT), along with malondialdehyde (MDA) concentration. Measurements were performed at a wavelength of 450 nm using a microplate ELISA reader as detailed in Ren et al. [53].

### 2.8. Histomorphological Examination

Following a 15-day bacterial challenge, fish intestine samples were fixed in 10% formalin for 48 h, dehydrated in graded ethanol, cleared in xylene, and paraffin-embedded. Five-micron sections were then cut using a microtome and stained with hematoxylin and eosin [54] for histological examination.

### 2.9. cDNA Synthesis and Real-Time PCR

Total RNA was extracted from the liver using QIAzol Lysis Reagent (QIAGEN, Hilden, Germany). The quality and quantity of the extracted RNA were confirmed by agarose gel electrophoresis and spectrophotometry, respectively. cDNA synthesis was performed using 2 µg of RNA, following the protocol of the FastLane Cell cDNA Kit (QIAGEN, Germany).

The expression of insulin-like growth factor 1 (*igf1*) and nuclear factor kappa B (*nf-κB*) was analyzed in the liver. Specific primers for these genes were designed using mRNA sequences available on the NCBI website. Details regarding primer sequences and corresponding gene bank accession numbers can be found in Table 2. To determine changes in gene expression levels between experimental groups, real-time PCR was performed using a Bio-RAD device (Milpitas, CA, USA) and SYBR green master mix (Enzynomics, Daejeon, Republic of Korea). The cycling conditions included 40 cycles of denaturation at 95 °C for 15 s, primer annealing at 60 °C for 1 min, and an extension step at 72 °C for 30 s. Amplification efficiencies were assessed using standard curve analysis. Based on these efficiencies, the modified 2^–ΔΔCt^ method was used to calculate relative changes in mRNA expression based on cycle threshold (Ct) values obtained during PCR. β-actin was used as the internal reference gene, following the method developed by Pfaffl [55] and briefly described by Elbialy et al. [56].

### 2.10. Statistical Analysis

After verifying data for normality and homogeneity of variance, statistical analysis was conducted using GraphPad Prism (version 8.01) software. One-way and two-way ANOVA tests were used to evaluate group differences, followed by Tukey’s post-hoc test for specific variations. Cumulative mortalities and relative protection were assessed using Kaplan–Meier survival analysis, with results interpreted by the log-rank (Mantel–Cox) test. Statistical significance was set at *p* < 0.05, indicated by different superscript letters. All results are presented as means ± SEM.

## 3. Results

### 3.1. Chemical Composition of MP Essential Oils

The major compounds in *Mentha piperita* (MP) were menthol and its derivative, levomenthol (Table 3). Levomenthol was the most abundant, accounting for 46.79% of the total detected compounds, followed by (-)-neomenthyl acetate (12.96%), L-menthone (5.77%), 2-cyclohexen-1-one, 3-methyl-6-(1-methylethyl)- (4.01%), and D-carvone (3.66%). Other notable compounds included cis-calamenene (3.22%), caryophyllene (3.02%), pulegone (1.25%), and eucalyptol (0.76%). The remaining compounds each contributed less than 2% to the total composition (Table 3).

### 3.2. Growth Performance, Feed Utilization, and Survivability

Fish fed diets supplemented with MP exhibited the highest growth performance compared to the control group after a 90-day experimental period (Table 4). Statistically significant improvements were observed across various growth metrics, including final body weight (FBW), weight gain (WG), average daily gain (ADG), specific growth rate (SGR), relative growth rate (RGR), final length, and length gain. The survival rates showed no significant differences among the groups, with no mortalities recorded during the 90-day experimental period. Interestingly, the 0.6% MP diet group achieved the highest numerical values for FBW, WG, ADG, SGR, RGR, final length, and length gain, while demonstrating the lowest FCR (Table 4).

### 3.3. Survivability

**Figure 1:**
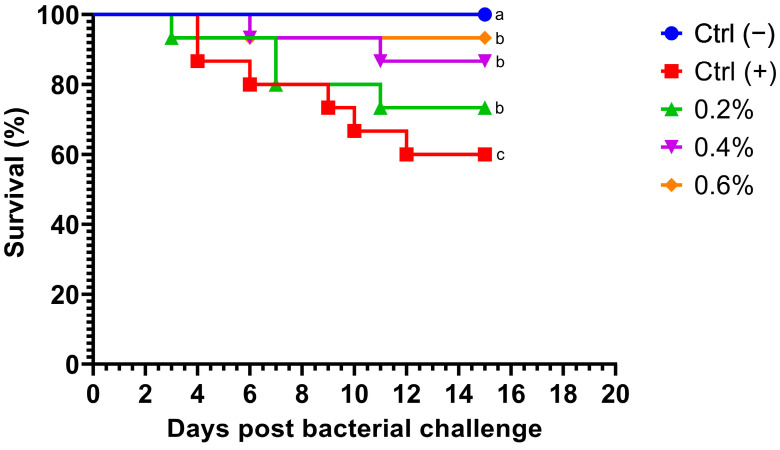
Kaplan–Meier survival curve of Nile tilapia (*Oreochromis niloticus*) fed diets supplemented with 0%, 0.2%, 0.4%, and 0.6% *Mentha piperita* (peppermint) for 90 days and subsequently challenged with *Aeromonas hydrophila*. Survival curves with different letters indicate significant differences between groups.

Fish fed MP at different concentrations exhibited a dramatic decrease in mortality after being challenged with *A. hydrophila*. Notably, no deaths occurred within the first 70 h post-challenge (Figure 1). Survival rates in fish challenged with the bacteria were significantly higher (*p* = 0.0254) in all treatment groups compared to the control group. Additionally, no mortalities were observed in the negative control group. Fish fed with the highest concentration of MP (0.6%) displayed the most significant cumulative survival rate compared to other treatments.

### 3.4. Hematology Results

Table 5 shows that dietary MP significantly impacted hematological parameters in fish both pre- and post-challenge assay. Fish fed MP diets exhibited slight but higher RBC and WBC counts compared to the control group, both before and after the challenge. Notably, the highest RBC and WBC counts were observed in the group fed 0.6% MP feed post-challenge. Hemoglobin (Hb) levels in both pre- and post-challenge groups also showed a significant increase in all MP-fed groups compared to the control. Importantly, all MP-fed groups demonstrated significantly higher packed cell volume (PCV) and mean corpuscular hemoglobin (MCH) compared to the control group.

Fish fed MP diets displayed altered proportions of lymphocytes, monocytes, neutrophils, and eosinophils compared to the control group. Although slight decreases in neutrophil levels were observed in the MP-fed groups prior to the challenge, this trend was reversed following the challenge. Lymphocyte levels increased both before and after the challenge in fish fed MP diets compared to the control. In contrast, monocytes and eosinophils showed minimal changes across all groups. The diet containing 0.6% MP demonstrated the most pronounced effect.

### 3.5. Biochemical Analyses

Table 6 highlights the impact of the MP diets on various biochemical parameters. Fish fed the MP diet, particularly at a 0.6% concentration, showed significant decreases in liver enzymes (ALT and AST) compared to the control group, both before and after the challenge. These enzymes increased post-challenge across all groups. Total protein and albumin content remained unaffected by the dietary treatments. Interestingly, serum globulin and triglycerides were significantly higher in the MP diet groups, especially at 0.6%, compared to the control, pre- and post-challenge. Additionally, the MP diet significantly reduced cholesterol, urea, and creatinine levels in fish both before and after the challenge.

### 3.6. Immune Responses

**Figure 2:**
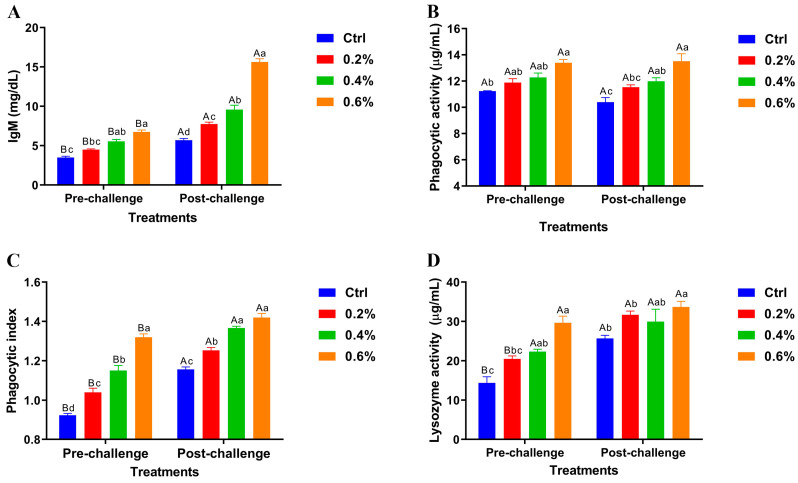
Pre- and post-challenge serum immunoglobulin M (IgM) levels (**A**), phagocytic activity (**B**), phagocytic index (**C**), and lysozyme activity (**D**) in Nile tilapia fed diets supplemented with 0%, 0.2%, 0.4%, and 0.6% *Mentha piperita* (peppermint) for 90 days and challenged with *Aeromonas hydrophila*. Data are expressed as the mean ± SEM. Values with different letter superscripts are significantly different between groups.

The results revealed significant variations in pre- and post-challenge levels of serum immunoglobulin M (IgM), phagocytic activity, phagocytic index, and lysozyme activity across the dietary groups shown in Figure 2A–D. Importantly, the highest MP concentration (0.6%) demonstrated highly significant differences in these immune parameters compared to the control and other treatment groups, both before and after the challenge.

### 3.7. Oxidative Stress Responses

The findings presented in Table 7 reveal a statistically significant increase in the antioxidant enzymes glutathione peroxidase (GPx), superoxide dismutase (SOD), and catalase (CAT) in fish fed diets supplemented with MP compared to the control, both before and after the challenge. Furthermore, the post-challenge readings for GPx, SOD, and CAT showed higher values compared to their respective pre-challenge levels. Conversely, the concentration of the oxidative stress marker malondialdehyde (MDA) was substantially lower in fish receiving MP supplementation, both pre- and post-challenge. Notably, the post-challenge MDA levels were lower than their pre-challenge levels in all groups, with 0.6% MP showing the lowest MDA activities.

### 3.8. Histomorphology Features

**Figure 3:**
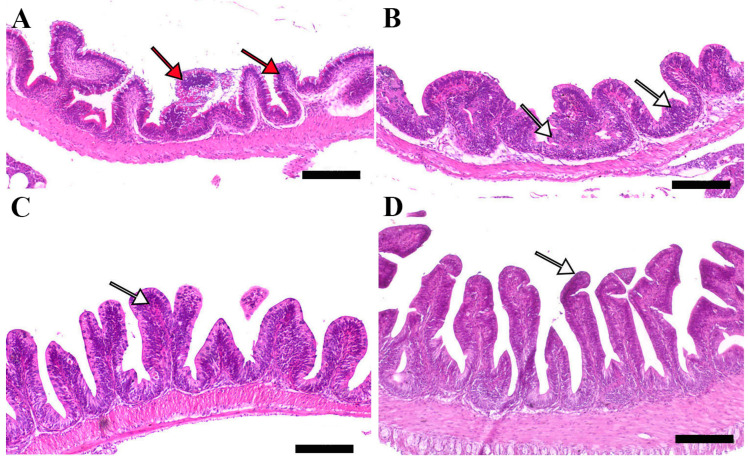
Representative histomorphological features (post-challenge) of H&E-stained sections from the anterior intestine of Nile tilapia fed diets supplemented with (**A**) 0%, (**B**) 0.2%, (**C**) 0.4%, or (**D**) 0.6% Mentha piperita (peppermint) for 90 days, followed by challenge with *Aeromonas hydrophila*. Red arrows indicate atrophic degenerative changes in the intestinal folds, while white arrows highlight normal intestinal folds. Scale bars = 100 μm.

**Figure 4:**
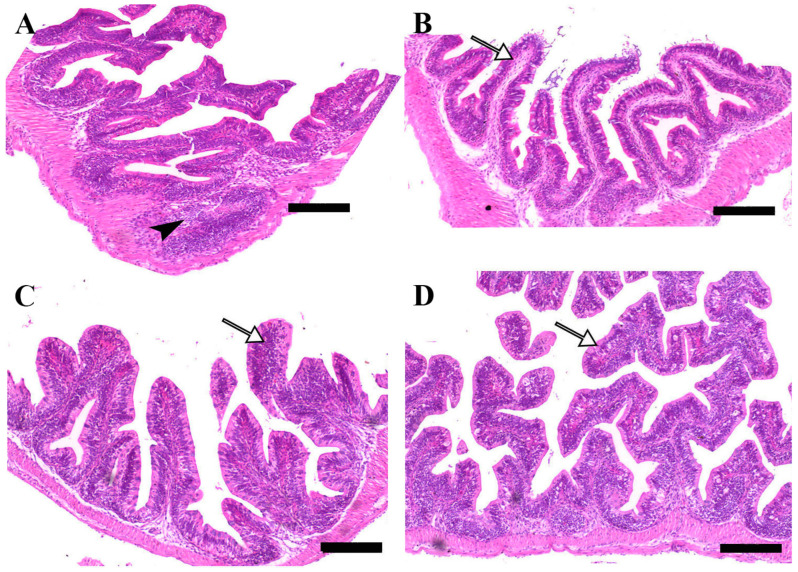
Representative histomorphological features of H&E-stained sections from the middle intestine of Nile tilapia fed diets supplemented with (**A**) 0%, (**B**) 0.2%, (**C**) 0.4%, or (**D**) 0.6% Mentha piperita (peppermint) for 90 days, followed by challenge with *Aeromonas hydrophila*. Black arrowheads indicate focal infiltration of inflammatory cells, while white arrows point to normal intestinal folds. Scale bars = 100 μm.

**Figure 5:**
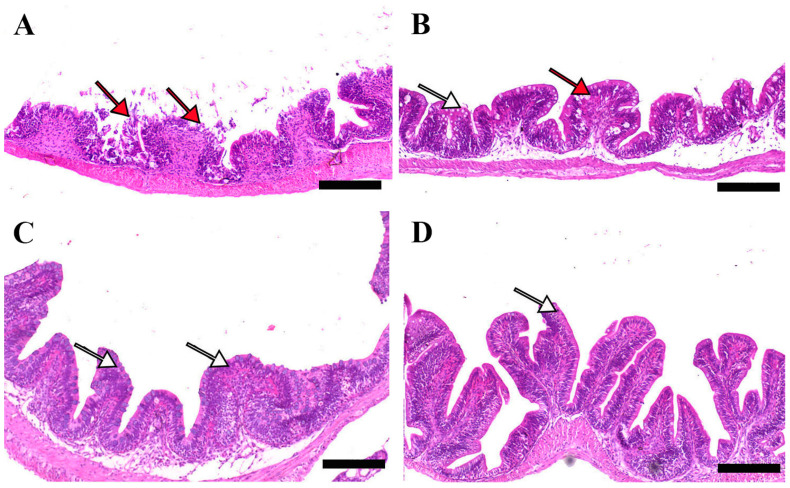
Representative histomorphological features of H&E-stained sections from the posterior intestine of Nile tilapia fed diets supplemented with (**A**) 0%, (**B**) 0.2%, (**C**) 0.4%, or (**D**) 0.6% Mentha piperita (peppermint) for 90 days, followed by challenge with Aeromonas hydrophila. Red arrows indicate atrophic degenerative changes in the intestinal folds, while white arrows highlight normal intestinal folds. Scale bars = 100 μm.

Microscopic examination of intestinal tissue sections after the challenge revealed atrophy, blunting of the mucosal folds, and signs of enteritis, including desquamation of the intestinal lining epithelium, in challenged fish receiving 0.0% MP (control). In contrast, fish supplemented with 0.2% and 0.4% dietary MP showed a reduction in these pathological changes. Notably, fish supplemented with 0.6% MP exhibited improved intestinal structural integrity and an increase in mucosal fold length, enhancing tissue stability against the bacterial challenge caused by *A. hydrophila* (Figure 3, Figure 4 and Figure 5).

### 3.9. Gene Expression Results

**Figure 6:**
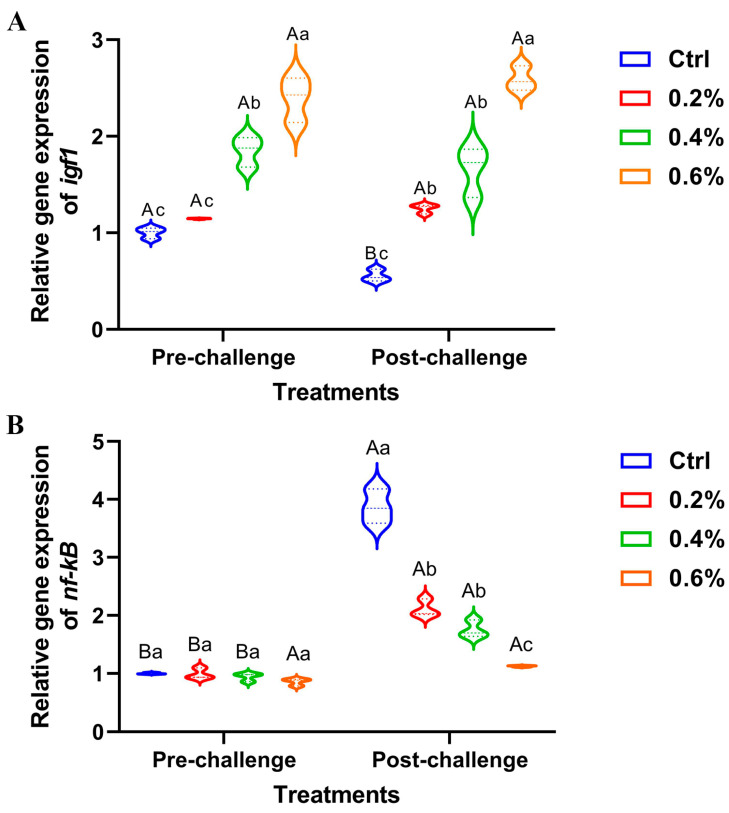
The expression of (**A**) insulin-like growth factor 1 (*igf1*) gene and (**B**) nuclear factor kappa B (*nf-κB*) gene in Nile tilapia fed diets supplemented with 0%, 0.2%, 0.4%, and 0.6% *Mentha piperita* (peppermint) for 90 days and challenged with *Aeromonas hydrophila*. Gene expression was normalized using *β*-actin as a reference gene. Data are expressed as the mean ± SEM. Values with distinct letter superscripts exhibit significant differences across groups.

The data presented in Figure 6A illustrates a significant upregulation of hepatic *igf1* expression, both pre- and post-challenge, in fish supplemented with MP, with the most pronounced effect observed in fish receiving 0.6%. There were no changes between the pre- and post-challenge stages for hepatic *igf1* expression. Conversely, the expression of the hepatic *nf-κB* gene exhibited non-significant downregulation in the pre-challenge stage, while significant upregulation was observed post-challenge, particularly in the control group compared to the other treatment groups (Figure 6B).

## 4. Discussion

MP is a versatile medicinal plant that offers numerous benefits in aquaculture [57]. It contains several potent properties and natural compounds that show a significant role in promoting the health and well-being of aquatic organisms [58]. MP, which contains 40–55% menthol, has antimicrobial effects and stress-reducing capabilities. It also has the potential to enhance the overall productivity and sustainability of aquaculture practice [59].

Our findings showed that Nile tilapia given diets containing MP for 90 days exhibited improved growth outcomes compared to the control group. The group with the MP concentration of 0.6% demonstrated favorable growth performance and the lowest FCR. These findings align with studies by Magouz, et al. [32] and Dawood, et al. [60], which highlighted how menthol essential oil extracted from peppermint plants improved the growth performance of Nile tilapia when included in their diet at ratios ranging from 0.2% to 0.3%.

The enhanced growth could be attributed to several factors. The results showed that MP upregulated the hepatic expression of *igf1*—an important gene in fish growth [61,62]. The *igf1* gene plays a critical role in regulating protein synthesis and muscle development in fish [63,64]. It stimulates muscle growth by suppressing protein breakdown and the expression of atrophy-related ubiquitin ligases, such as atrogin-1 and muscle ring finger 1 [65]. Phytogenic feed additives derived from herbs have been documented to enhance the expression of growth- and immune-related genes, including *gh* and *igf1* [18]. These additives may influence growth and other physiological pathways either by directly stimulating gene expression or by enhancing other growth-related factors such as intestinal health, nutrient absorption, and feed utilization. Additionally, they help minimize environmental stress, creating conditions conducive to normal physiological functioning, which in turn promotes growth and overall health [66,67].

The observed enhancement in the growth performance of Nile tilapia fed MP-supplemented diets can be attributed to MP’s ability to boost the activity of digestive enzymes, thereby improving digestion and absorption, as suggested by Aguiar, et al. [27]. Dietary phytogenic additives have consistently been shown to increase digestive enzyme activity in fish [68,69]. This improved feed utilization translates into increased growth rates and reduced FCR [70]. Additionally, mint plants consist of substances such as menthol, which may improve the taste of the feed, encouraging higher consumption and ultimately better growth [71]. Thus, the mint plant could act as a potential natural growth enhancer in Nile tilapia farming, supporting output and financial gains.

Regarding the effect on fish health, *A. hydrophila* is a major pathogen causing severe economic strain in tilapia aquaculture, leading to high mortality rates [72,73]. Evaluating a fish’s tolerance to such infections is essential for understanding the benefits of dietary additives [74]. Similar to the established effectiveness of MP supplementation as an antimicrobial agent [25,75], this study found that fish fed MP-supplemented diets exhibited significantly higher survival rates compared to the control group after bacterial challenge. Notably, no mortalities were observed in the negative control group, and fish fed the highest MP concentration (0.6%) displayed the highest cumulative survival rate.

The resistance of Nile tilapia fed MP could be attributed to the various bioactive components present in the plant. Peppermint contains essential oils (e.g., menthol, levomenthol and menthone) and phenolic compounds with known antimicrobial and immunomodulatory properties [76,77]. Menthol disrupts the bacterial communication system (quorum sensing; QS) in various Gram-negative pathogens, including *A. hydrophila* [78]. These pathogens utilize diverse acyl homoserine lactone (AHL) molecules for QS. Menthol’s effect was observed through a reduction in AHL-dependent production of violacein (a pigment), virulence factors, and biofilm formation, suggesting broad-spectrum anti-QS activity [78].

Fish fed MP-supplemented diets significantly improved blood parameters compared to the control group in both pre- and post-bacterial challenge groups. These improvements included higher RBC and WBC counts, increased Hb percentage, PCV, and MCH. Additionally, MP influenced the proportions of WBC populations, including lymphocytes, monocytes, neutrophils, and eosinophils. Similarly, Nile tilapia fed 2% MP showed significantly enhanced hematological parameters compared to those fed 3% and 4% MP [23]. Moreover, a concentration of 0.5% MP exhibited a higher antibacterial effect after infection by *Streptococcus agalactiae* in red tilapia [79]. The composition of MP (e.g., menthol, menthone, and menthofuran) may promote the production of red and white blood cells in the fish. Increased Hb levels suggest better oxygen delivery to tissues, indicating the ability of MP to improve oxygen transport capacity [80,81]. In this way, MP shows a mitigating function against the degenerative effects that may follow infection.

Fish fed MP-supplemented diets, particularly at a 0.6% concentration, exhibited significant reductions in liver enzymes (ALT and AST) as well as cholesterol, urea, and creatinine levels, both before and after a bacterial challenge. This is noteworthy, as *Aeromonas hydrophila* infection is known to cause hepatic and renal damage in fish [73,82], leading to elevated liver enzymes, urea, and creatinine levels [83]. The reductions observed in these parameters in MP-fed fish indicate improved hepatic and renal function, as well as overall health.

Menthol, the major component of MP, has been found to protect against sepsis-induced hepatic injury [84]. It significantly reduces serum liver enzyme levels and hepatic concentrations of TNF-α, MDA, and cleaved caspase-3 while maintaining balanced hepatic SOD and GSH levels. Additionally, menthol enhances biomarkers associated with regeneration and survival, such as B-cell lymphoma 2 (an anti-apoptotic factor) and proliferating cell nuclear antigen, following sepsis-induced liver injury. These effects contribute to improved hepatic histopathological changes [84]. Moreover, menthol can modulate inflammatory molecules, including Toll-like receptor 4, myeloid differentiation primary response 88, and NF-κB, to protect against liver and brain injuries [85]. MP also exhibits renal protective effects by reducing lipid peroxidation, as well as urea and creatinine levels, in injured kidneys [86].

Furthermore, menthone, a component of MP, possesses both local and systemic anti-inflammatory properties [87]. It regulates type-I interferon signaling through Tyk2 ubiquitination to modulate local inflammation [88], influences T-cell subtypes, and reduces pro-inflammatory cytokines [89]. Additionally, menthone has demonstrated protective effects against DNA damage [90]. Both menthol and menthone exhibit protective and anti-inflammatory properties against parasite-induced injury in the liver and intestine [91]. Consequently, menthone may offer protective benefits for the gastrointestinal tract and other internal organs.

Furthermore, MP-fed groups displayed higher serum globulin and triglyceride levels, while total protein and albumin levels remained unchanged. Similar findings have been reported in rohu (*Labeo rohita*) fingerlings, where lower cholesterol and glucose levels were observed alongside enhanced resistance to *A. hydrophila* [37]. Serum globulin primarily consists of immunoglobulins [92], and its increase suggests enhanced humoral immune activity and elevated immunoglobulin levels in MP-fed fish. Additionally, reduced cholesterol levels in MP-fed fish may have further supported their immune response, as elevated cholesterol is known to impair normal immune function in fish [93]. Dietary cholesterol has been shown to induce inflammation by upregulating pro-inflammatory cytokine expression while suppressing anti-inflammatory cytokines, partly through the modulation of NF-κB and TOR signaling pathways in fish immune organs [93].

MP contains various concentrations of limonene, pulegone, carvone, and eucalyptol (1,8-cineole), which contribute to its beneficial biological activities [94]. Limonene has anti-inflammatory properties by inhibiting the NF-κB/AP-1 pathway [95]. Carvone has potent antipathogenic effects (e.g., antibacterial, antifungal, antiparasitic) as well as antistress effects [96].

Other active compounds detected in MP, such as caryophyllene, pulegone, and eucalyptol, also exhibit significant biological activity. These compounds demonstrate antibacterial effects by altering membrane permeability and integrity in various bacteria, such as *Bacillus cereus*, leading to membrane damage [97,98,99]. Caryophyllene additionally possesses antioxidant and anti-inflammatory properties [100]. Pulegone suppresses the expression of biofilm-formation-related genes in bacteria such as *Escherichia coli* [101]. Eucalyptol has antioxidant, antimicrobial, and pro-apoptotic effects, and exerts its anti-inflammatory activity by suppressing NF-κB p65 [102].

Consistent with these characteristics, the results indicate that MP significantly downregulates hepatic *nf-κB* expression. Furthermore, reductions in ALT and AST levels suggest that MP may provide hepatoprotective effects through its anti-inflammatory and antioxidant properties [103]. Additionally, the observed decrease in urea and creatinine levels implies that MP may support kidney function by enhancing waste product removal [35]. These findings suggest that dietary MP can positively influence fish health and metabolism by improving their biochemical parameters.

Dietary MP improved serum IgM levels, phagocytic, and lysozyme activities in a dose-dependent manner, with the best performance at the concentration of 0.6%. These improvements were observed both before and after the bacterial challenge. The bactericidal activity of MP stems from the presence of flavonoids, tannins, and other bioactive compounds. MP is also a good source of vitamins A and C, as well as minerals like potassium and calcium, which are important for enhancing the immune system [104,105]. MP’s bioactive components help scavenge free radicals, mitigating oxidative stress and improving overall health [106,107]. MP significantly enhances antioxidant defenses—as evidenced by increased GPx, SOD, and CAT levels—while decreasing MDA, suggesting advanced resilience towards oxidative stress and protection against infections.

This study revealed that dietary supplementation with MP, particularly at the concentration of 0.6%, protected the intestinal integrity of fish challenged with *A. hydrophila*. MP promoted beneficial bacteria, such as *Lactobacillus* and *Bifidobacterium* [108], suggesting its contribution to achieving a balanced gut microbiota, thereby improving gut and immune health. Furthermore, MP can act as a prebiotic compound [109], providing nourishment for beneficial gut bacteria and supporting their proliferation.

These findings suggest that MP can be a promising natural strategy to improve gut health and protect against intestinal damage caused by bacterial infections in fish. However, further research is needed to explore the underlying mechanisms, including the expression of additional immune- and growth-related genes, changes in immune effectors such as complement activity, as well as transcriptomic and microbiota alterations.

## 5. Conclusions

*Mentha piperita* (MP) can contribute to improved fish health and performance in aquaculture. This study provides compelling evidence for the potential of MP as a valuable medicinal plant in aquaculture, specifically as a dietary supplement for Nile tilapia. Using MP could be environmentally friendly, minimizing reliance on antibiotics. Future research should aim to determine the optimal dose and methods of delivering MP, provide a more detailed description of the mechanisms by which it works, and evaluate the effectiveness of MP against different pathogens and in different fish species. Additionally, investigating possible interactions with other natural feed additives could help develop multi-faceted feeding plans to enhance fish health and output in aquaculture.

## Figures and Tables

**Table 1:** Feed ingredients and proximate analysis of diets.

Ingredients	Experimental Diets (%)			
	Ctrl	0.2%	0.4%	0.6%
Soybean meal	37	37	37	37
Fishmeal (72% CP)	4	4	4	4
Yellow corn	19.4	19.4	19.4	19.4
Fish oil	0.7	0.7	0.7	0.7
Soy oil	0.7	0.7	0.7	0.7
Rice bran	11.2	11.2	11.2	11.2
Wheat bran	16.5	16.3	16.1	15.9
Corn gluten	6	6	6	6
Di calcium phosphate	0.6	0.6	0.6	0.6
Premix ^1^	3.9	3.9	3.9	3.9
MP ^2^	0	0.2	0.4	0.6
Chemical analysis (% DM basis)				
Dry matter	90.46	90.51	90.36	90.46
Crude protein	30.04	30.10	30.11	30.13
Ether extract	6.14	6.36	6.57	6.79
Fiber	5.11	5.13	5.10	5.9
Ash	6.11	6.12	6.13	6.17
Carbohydrate	43.06	42.8	42.45	41.47
Gross energy (KJ/g) ^3^	18.38	18.43	18.49	18.38

^1^ Premix content of vitamins and minerals was detailed by Ashry, et al. [41]. ^2^ MP, Mentha piperita. ^3^ Gross energy was calculated based on the values for protein, lipid, and carbohydrate as 23.6, 39.5, and 17.2 KJ/g, respectively.

**Table 2:** Primers used for real-time PCR.

Gene	Primer Sequence (5′-3′)		GenBank Accession No.
*igf1*	Forward	CACCCTCTCACTACTGCTGT	EU272149.1
	Reverse	TCGCTCTCCACAGACAAACT	
*nf-kB*	Forward	GCAGTCACAACCACAGCAAT	XM_025910842.1
	Reverse	TCCCCAAACTTCAGGACAAC	
*β*-actin	Forward	TAATAACAGAACGCAGCGCC	EU887951.1
	Reverse	AGTGCGGCGATTTCATCTTC	

Insulin-like growth factor 1 (*igf1*); nuclear factor kappa B (*nf-κB*).

**Table 3:** GC/Mass of *M. piperita* essential oils.

Peak	Retention Time	Name	Formula	Area	Area Sum %
1	1.189	Cyclohexanol, 4-methyl-, trans-	C_7_H_14_O	14,973,414.1	1.05
2	4.654	Eucalyptol	C_10_H_18_O	10,851,421.8	0.76
3	7.557	L-Menthone	C_10_H_18_O	82,332,535.7	5.77
4	7.888	Cyclohexanol, 5-methyl-2-(1-methylethyl)-	C_10_H_20_O	11,657,418.3	0.82
5	8.683	Levomenthol	C_10_H_20_O	667,724,412	46.79
6	9.727	Pulegone	C_10_H_16_O	17,769,949.4	1.25
7	10.059	D-Carvone	C_10_H_14_O	52,251,070.7	3.66
8	10.315	2-Cyclohexen-1-one, 3-methyl-6-(1-methylethyl)-	C_10_H_16_O	57,150,900.4	4.01
9	10.647	2H-1-Benzopyran, 3,4,4a,5,6,8a-hexahydro-2,5,5,8a-tetramethyl-, (2.alpha.,4a.alpha.,8a.alpha.)-	C_13_H_22_O	20,616,656.6	1.44
10	10.947	(-)-Neomenthyl acetate	C_12_H_22_O_2_	184,890,792	12.96
11	11.316	Phenol, 2-methyl-5-(1-methylethyl)-	C_10_H_14_O	12,522,942.1	0.88
12	12.805	(-)-.beta.-Bourbonene	C_15_H_24_	19,837,543.6	1.39
13	13.03	Cyclohexane, 1-ethenyl-1-methyl-2,4-bis(1-methylethenyl)-, [1S-(1.alpha.,2.beta.,4.beta.)]-	C_15_H_24_	23,508,773	1.65
14	13.324	Bicyclo[5.2.0]nonane, 2-methylene-4,8,8-trimethyl-4-vinyl-	C_15_H_24_	10,008,891.2	0.7
15	13.725	Caryophyllene	C_15_H_24_	43,141,424	3.02
16	15.282	Naphthalene, 1,2,3,5,6,7,8,8a-octahydro-1,8a-dimethyl-7-(1-methylethenyl)-, [1S-(1.alpha.,7.alpha.,8a.alpha.)]-	C_15_H_24_	11,481,210.5	0.8
17	15.345	Ylangenol	C_15_H_24_O	8,163,010.15	0.57
18	15.476	Naphthalene, decahydro-4a-methyl-1-methylene-7-(1-methylethylidene)-, (4aR-trans)-	C_15_H_24_	16,145,401.3	1.13
19	16.114	cis-Calamenene	C_15_H_22_	45,993,717.8	3.22
20	17.522	1H-Cycloprop[e]azulen-7-ol, decahydro-1,1,7-trimethyl-4-methylene-, [1ar-(1a.alpha.,4a.alpha.,7.beta.,7a.beta.,7b.alpha.)]-	C_15_H_24_O	50,757,939.2	3.56
21	17.847	1H-Cycloprop[e]azulen-4-ol, decahydro-1,1,4,7-tetramethyl-, [1aR-(1a.alpha.,4.beta.,4a.beta.,7.alpha.,7a.beta.,7b.alpha.)]-	C_15_H_26_O	23,831,854.8	1.67
22	18.247	4a(2H)-Naphthalenol, 1,3,4,5,6,8a-hexahydro-4,7-dimethyl-1-(1-methylethyl)-, (1S,4R,4aS,8aR)-	C_15_H_26_O	9,229,174.01	0.65
23	19.198	beta.-Guaiene	C_15_H_24_	8,637,204.79	0.61
24	19.267	2-((2R,4aR,8aS)-4a-Methyl-8-methylenedecahydronaphthalen-2-yl)prop-2-en-1-ol	C_15_H_24_O	7,996,558.6	0.56
25	22.789	2-Pentadecanone, 6,10,14-trimethyl-	C_18_H_36_O	15,444,721.8	1.08

**Table 4:** Growth performance parameters of Nile tilapia fed different dietary levels of *Mentha piperita* (peppermint)-supplemented diets for 90 days.

Items	Treatments				*p* Value
	Ctrl	0.2%	0.4%	0.6%	
IBW (g)	10.87 ± 0.078	10.85 ± 0.096	10.75 ± 0.023	10.84 ± 0.060	0.6757
FBW (g)	37.40 ± 1.222 ^c^	43.22 ± 1.636 ^b^	46.65 ± 0.096 ^b^	51.67 ± 0.540 ^a^	0.0001
WG (g)	26.53 ± 1.163 ^c^	32.38 ± 1.725 ^b^	35.89 ± 0.117 ^b^	40.82 ± 0.560 ^a^	0.0001
ADG (g/fish/day)	0.2948 ± 0.013 ^c^	0.3598 ± 0.019 ^b^	0.3988 ± 0.001 ^b^	0.4536 ± 0.006 ^a^	0.0001
SGR (%/day)	1.372 ± 0.031 ^c^	1.535 ± 0.052 ^b^	1.630 ± 0.005 ^ab^	1.735 ± 0.015 ^a^	0.0002
RGR (%)	344.1 ± 9.604 ^c^	398.9 ± 18.29 ^b^	433.7 ± 1.754 ^ab^	476.5 ± 6.278 ^a^	0.0002
Initial length (cm)	8.937 ± 0.3044	9.113 ± 0.1462	8.607 ± 0.8399	8.933 ± 0.3034	0.8962
Final length (cm)	14.63 ± 0.055 ^d^	15.46 ± 0.090 ^c^	16.29 ± 0.162 ^b^	17.01 ± 0.227 ^a^	0.0001
Length gain (cm)	5.697 ± 0.343 ^b^	6.343 ± 0.074 ^ab^	7.687 ± 0.742 ^a^	8.080 ± 0.091 ^a^	0.0109
K	1.197 ± 0.048	1.170 ± 0.042	1.080 ± 0.029	1.053 ± 0.045	0.1117
FCR (g)	1.973 ± 0.091 ^a^	1.620 ± 0.092 ^b^	1.453 ± 0.003 ^bc^	1.277 ± 0.018 ^c^	0.0004

IBW, initial body weight; FBW, final body weight; WG, weight gain; ADG, average daily gain; SGR, specific growth rate; RGR, relative growth rate; K, condition factor; FCR, feed conversion factor. Values are presented as mean ± SEM. Different letters indicate significance.

**Table 5:** Hematological indices (mean ± SEM) of Nile tilapia fed different dietary levels of *Mentha piperita* (peppermint)-supplemented diets for 90 days.

Parameters	Pre- and Post-Challenge	Treatments				*p* Value		
		Ctrl	0.2%	0.4%	0.6%	Treatment	Challenge Test	Interaction
RBCs (×10^3^/mm^3^)	Before	2.210 ± 0.036 ^Bc^	2.447 ± 0.009 ^Ab^	2.603 ± 0.035 ^Bb^	2.797 ± 0.023 ^Ba^	0.0001	0.0001	0.0429
	After	2.380 ± 0.035 ^Ac^	2.570 ± 0.049 ^Ab^	2.930 ± 0.029 ^Aa^	3.033 ± 0.038 ^Aa^			
Hb (g/100 mL)	Before	7.270 ± 0.038 ^Bc^	7.560 ± 0.047 ^Bc^	8.147 ± 0.105 ^Ab^	8.497 ± 0.090 ^Aa^	0.0001	0.0001	0.0488
	After	7.797 ± 0.027 ^Ac^	8.047 ± 0.078 ^Abc^	8.330 ± 0.055 ^Ab^	8.737 ± 0.063 ^Aa^			
PCV (%)	Before	24.767 ± 0.055 ^Bc^	25.673 ± 0.339 ^Bc^	27.703 ± 0.274 ^Bb^	28.690 ± 0.067 ^Ba^	0.0001	0.0001	0.0001
	After	28.313 ± 0.043 ^Ac^	28.973 ± 0.196 ^Abc^	29.350 ± 0.200 ^Aab^	30.150 ± 0.102 ^Aa^			
MCH	Before	28.757 ± 0.315 ^Ab^	29.280 ± 0.040 ^Ab^	29.763 ± 0.348 ^Aab^	30.510 ± 0.366 ^Aa^	0.0001	0.1728	0.8006
	After	29.270 ± 0.070 ^Ab^	29.450 ± 0.064 ^Ab^	29.823 ± 0.141 ^Aab^	30.723 ± 0.254 ^Aa^			
WBCs (×10^3^/mm^3^)	Before	12.300 ± 0.491 ^Bc^	15.977 ± 0.348 ^Bb^	16.747 ± 0.356 ^Bab^	18.220 ± 0.445 ^Ba^	0.0001	0.0001	0.8606
	After	16.237 ± 0.574 ^Ac^	20.010 ± 0.340 ^Ab^	20.590 ± 0.431 ^Ab^	22.737 ± 0.348 ^Aa^			
Neutrophil (×10^3^/mm^3^)	Before	1.357 ± 0.015 ^Aa^	1.080 ± 0.015 ^Bb^	1.020 ± 0.006 ^Bb^	1.073 ± 0.012 ^Bb^	0.0001	0.0001	0.0001
	After	1.333 ± 0.009 ^Ac^	2.050 ± 0.025 ^Aa^	1.823 ± 0.097 ^Ab^	2.067 ± 0.034 ^Aa^			
Lymphocyte (×10^3^/mm^3^)	Before	7.870 ± 0.060 ^Bc^	8.560 ± 0.068 ^Bb^	8.763 ± 0.073 ^Bab^	8.947 ± 0.119 ^Ba^	0.0001	0.0001	0.0084
	After	9.137 ± 0.037 ^Ac^	9.840 ± 0.079 ^Ab^	9.687 ± 0.064 ^Ab^	10.500 ± 0.093 ^Aa^			
Monocyte (×10^3^/mm^3^)	Before	1.003 ± 0.033 ^Ab^	1.153 ± 0.038 ^Aa^	1.117 ± 0.042 ^Aab^	1.200 ± 0.012 ^Aa^	0.0001	0.0270	0.7611
	After	1.047 ± 0.024 ^Ab^	1.227 ± 0.027 ^Aa^	1.183 ± 0.029 ^Aab^	1.217 ± 0.012 ^Aa^			
Eosinophil (×10^3^/mm^3^)	Before	0.130 ± 0.012	0.127 ± 0.015	0.123 ± 0.007	0.130 ± 0.006	0.9687	0.7369	0.8805
	After	0.133 ± 0.004	0.130 ± 0.010	0.133 ± 0.018	0.123 ± 0.003			

RBCs, red blood cells; Hb, hemoglobin; PCV, Packed cell volume; MCH, Mean corpuscular hemoglobin; WBCs, white blood cells. Different superscript letters indicate significant treatment differences (*p* < 0.05). Lowercase letters indicate significant differences between experimental groups, while uppercase letters indicate significant differences between pre- and post-challenge.

**Table 6:** Biochemical analysis (mean ± SEM) of Nile tilapia fed different dietary levels of *Mentha piperita* (peppermint)-supplemented diets for 90 days.

Parameters	Pre- and Post-Challenge	Treatments				*p* Value		
		Ctrl	0.2%	0.4%	0.6%	Treatment	Challenge Test	Interaction
ALT (U/L)	Before	28.413 ± 0.732 ^Ba^	27.173 ± 0.099 ^Ba^	26.550 ± 0.053 ^Ba^	25.370 ± 0.142 ^Ba^	0.0001	0.0001	0.0001
	After	52.040 ± 0.880 ^Aa^	44.943 ± 0.894 ^Ab^	40.817 ± 0.691 ^Ac^	35.680 ± 0.981 ^Ad^			
AST (U/L)	Before	31.443 ± 0.669 ^Ba^	27.770 ± 1.194 ^Ba^	24.590 ± 0.645 ^Bb^	24.593 ± 0.906 ^Bb^	0.0001	0.0001	0.0001
	After	56.903 ± 0.779 ^Aa^	54.510 ± 0.575 ^Aa^	46.140 ± 1.372 ^Ab^	34.397 ± 1.510 ^Ac^			
TP (g/dL)	Before	4.147 ± 0.292	4.303 ± 0.212	4.043 ± 0.244	4.633 ± 0.197	0.3270	0.0832	0.6363
	After	4.460 ± 0.220	4.623 ± 0.122	4.497 ± 0.117	4.587 ± 0.109			
Albumin (g/dL)	Before	1.382 ± 0.027	1.435 ± 0.030	1.402 ± 0.031	1.410 ± 0.016	0.5597	0.7430	0.6027
	After	1.389 ± 0.034	1.398 ± 0.033	1.442 ± 0.028	1.427 ± 0.026			
Globulin (g/dL)	Before	1.760 ± 0.029 ^Bd^	2.130 ± 0.017 ^Bc^	2.270 ± 0.055 ^Ab^	2.513 ± 0.034 ^Aa^	0.0001	0.0001	0.0163
	After	2.013 ± 0.019 ^Ac^	2.273 ± 0.020 ^Ab^	2.337 ± 0.009 ^Ab^	2.590 ± 0.015 ^Aa^			
Triglyceride (mg/dL)	Before	87.740 ± 0.577 ^Ac^	90.840 ± 0.263 ^Ab^	93.257 ± 0.667 ^Ab^	97.377 ± 0.546 ^Aa^	0.0001	0.0038	0.6549
	After	89.297 ± 0.604 ^Ac^	91.520 ± 0.416 ^Ac^	95.190 ± 0.329 ^Ab^	98.350 ± 0.726 ^Aa^			
Cholesterol (mg/dL)	Before	117.450 ± 3.473 ^Aa^	98.923 ± 0.980 ^Ab^	92.137 ± 1.235 ^Ab^	89.923 ± 0.997 ^Ab^	0.0001	0.0027	0.3450
	After	126.327 ± 3.542 ^Aa^	110.690 ± 5.539 ^Ab^	96.620 ± 0.741 ^Ac^	92.313 ± 0.965 ^Ac^			
Urea (mg/dL)	Before	4.243 ± 0.344 ^Ba^	3.947 ± 0.262 ^Ba^	4.003 ± 0.235 ^Ba^	3.863 ± 0.372 ^Ba^	0.9892	0.0001	0.9783
	After	7.443 ± 0.514 ^Aa^	7.483 ± 0.696 ^Aa^	7.323 ± 0.387 ^Aa^	7.513 ± 1.156 ^Aa^			
Creatinine (mg/dL)	Before	0.450 ± 0.006 ^Ba^	0.470 ± 0.032 ^Ba^	0.377 ± 0.024 ^Ba^	0.440 ± 0.025 ^Ba^	0.2733	0.0001	0.6516
	After	1.547 ± 0.173 ^Aa^	1.440 ± 0.0115 ^Aa^	1.253 ± 0.176 ^Aa^	1.530 ± 0.127 ^Aa^			

ALT, alanine aminotransferase; AST, aspartate aminotransferase; and TP, total protein. Different superscript letters indicate significant differences (*p* < 0.05). Lowercase letters indicate significant differences between experimental groups, while uppercase letters indicate significant differences between pre- and post-challenge.

**Table 7:** Antioxidant analysis (mean ± SEM; nmol/mL) of Nile tilapia fed different dietary levels of *Mentha piperita* (peppermint)-supplemented diets for 90 days.

Parameters	Pre- and Post-Challenge	Treatments				*p* Value		
		Ctrl	0.2%	0.4%	0.6%	Treatment	Challenge Test	Interaction
MDA	Before	10.707 ± 0.539 ^Ba^	8.870 ± 0.137 ^Bab^	6.460 ± 0.462 ^Bbc^	4.593 ± 0.358 ^Bc^	0.0001	0.0001	0.0019
	After	19.333 ± 0.860 ^Aa^	12.280 ± 0.859 ^Ab^	12.643 ± 0.205 ^Ab^	11.010 ± 0.390 ^Ab^			
CAT	Before	35.110 ± 1.683 ^Bc^	45.817 ± 1.316 ^Bb^	47.967 ± 1.372 ^Bb^	61.530 ± 1.580 ^Ba^	0.0001	0.0001	0.0127
	After	67.360 ± 1.251 ^Ab^	69.410 ± 0.548 ^Ab^	74.437 ± 2.221 ^Ab^	83.200 ± 1.187 ^Aa^			
Gpx	Before	34.940 ± 0.726 ^Bc^	41.506 ± 0.948 ^Bb^	44.823 ± 0.397 ^Bab^	47.803 ± 1.046 ^Ba^	0.0001	0.0001	0.1149
	After	51.443 ± 1.133 ^Ac^	54.513 ± 1.102 ^Abc^	58.283 ± 0.489 ^Aab^	60.363 ± 0.321 ^Aa^			
SOD	Before	32.233 ± 1.511 ^Ab^	41.047 ± 2.667 ^Aab^	45.776 ± 0.773 ^Aab^	48.967 ± 1.161 ^Aa^	0.0002	0.0234	0.4663
	After	34.960 ± 4.789 ^Ab^	52.653 ± 3.431 ^Aa^	48.153 ± 3.093 ^Aab^	54.947 ± 5.131 ^Aa^			

MDA, malondialdehyde; CAT, catalase; GPx, glutathione peroxidase; SOD, superoxide dismutase. Different superscript letters indicate significant differences (*p* < 0.05). Lowercase letters indicate significant differences between experimental groups, while uppercase letters indicate significant differences between pre- and post-challenge.

## Data Availability

The original contributions presented in this study are included in the article. Further inquiries can be directed to Ahmed E. Elshafey (ahmed_alshafei@fsh.kfs.edu.eg) upon reasonable request.

## Abbreviations

The following abbreviations are used in this manuscript:

ADG	average daily gain
AHL	acyl homoserine lactone
ALT	alanine aminotransferase
AST	aspartate aminotransferase
CAT	catalase
FBW	final body weight
FCR	feed conversion ratio
GPx	glutathione peroxidase
Hb	hemoglobin
igf1	insulin-like growth factor 1
IgM	immunoglobulin M
K	condition factor
MCH	mean corpuscular hemoglobin
MDA	malondialdehyde
MP	mentha piperita
nf-κB	nuclear factor kappa B
PCV	packed cell volume
RBCs	red blood cells
RGR	relative growth rate
SGR	specific growth rate
SOD	superoxide dismutase
WBC	white blood cell
WG	weight gain ratio
